# How immigrants adapt their smoking behaviour: comparative analysis among Turkish immigrants in Germany and the Netherlands

**DOI:** 10.1186/1471-2458-14-844

**Published:** 2014-08-14

**Authors:** Katharina Reiss, Odile Sauzet, Jürgen Breckenkamp, Jacob Spallek, Oliver Razum

**Affiliations:** Department of Epidemiology & International Public Health, Bielefeld School of Public Health (BiSPH), Bielefeld University, P.O. Box 10 01 31, 33501 Bielefeld, Germany

**Keywords:** Smoking, Emigrants and immigrants, Comparative study, Netherlands, Germany, Length of stay

## Abstract

**Background:**

Smoking behaviour among immigrants is assumed to converge to that of the host country’s majority population with increasing duration of stay. We compared smoking prevalence among Turkish immigrants residing in two different countries (Germany (DE)/the Netherlands (NL)) between and within countries by time spent in Turkey and DE/NL.

**Methods:**

The German 2009 micro-census and the Dutch POLS database (national survey, 1997–2004) were analysed. An interaction variable with dichotomised length of stay (LOS) in Turkey (age: 0–17; 18+) and categorised LOS in the host country (immigration year: 1979 and earlier, 1980–1999, 2000–2009; the latter only for Germany) was generated. Age standardised smoking prevalences and sex-specific logistic regression models were calculated.

**Results:**

6,517 Turkish participants were identified in Germany, 2,106 in the Netherlands. Age-standardised smoking prevalences were higher among Turkish immigrants in the Netherlands compared to those in Germany: 62.3% vs. 53.1% (men/lower education); 30.6% vs. 23.0% (women/lower education). A similar trend was observed for the majority population of both countries. The chance of being a smoker was lower among Turkish men with short LOS in Turkey and middle LOS in Germany/the Netherlands compared to those with short LOS in Turkey and long LOS in Germany/the Netherlands (NL: OR = 0.57[95% CI = 0.36-0.89]; DE: OR = 0.73[95% CI = 0.56-0.95]). Contrary to that, the chance of being a smoker was higher among Turkish men with long LOS in Turkey and middle LOS in Germany/the Netherlands compared to those with long LOS in Turkey and long LOS in Germany/the Netherlands (NL: OR = 1.35[95% CI = 0.79-2.33]; DE: OR = 1.44[95% CI = 1.03-2.02]). The effects for Turkish women were similar, but smaller and often non-significant.

**Conclusion:**

Turkish immigrants adapt their smoking behaviour towards that of the Dutch/German majority population with increasing duration of stay. This was particularly obvious among those who left Turkey before the age of 18 years – a group that needs tailored interventions to prevent further increases in smoking. Those who left Turkey as adults and spent a short time in the host countries show ‘imported’ smoking patterns. A limitation of this study is the use of cross-sectional data: a cohort effect cannot be ruled out. Our findings have to be confirmed with longitudinal data.

## Introduction

Smoking is one of the leading causes for various cancers (e.g. lung, oral, pancreas or stomach cancer) and cardiovascular diseases (e.g. atherosclerosis or myocardial infarction) [1–3]. It is known to be strongly associated with the socioeconomic position at the individual level but may also be influenced by political tobacco control efforts at the macro level [4, 5]. While smoking behaviour in general is well studied, research is scarce in terms of smoking among persons with a personal immigration experience. Here, the question arises of whether and how immigrants *adapt* to the smoking behaviour of the majority population of a country.

In 2011, 1.8% (n = 1,491,000) of the population of Germany and 1.2% (n = 197,042) of the population of the Netherlands were born in Turkey [6, 7]. Given the special situation of a population that emigrated from the same country of origin and resides in two different host countries, a comparison of the smoking behaviour between Turkish immigrants now residing in Germany and the Netherlands would reveal a possible societal influence of the different host countries. As current smoking might also be influenced by the society of the country of origin, it is important to take this aspect into account as well.

**Figure 1:**
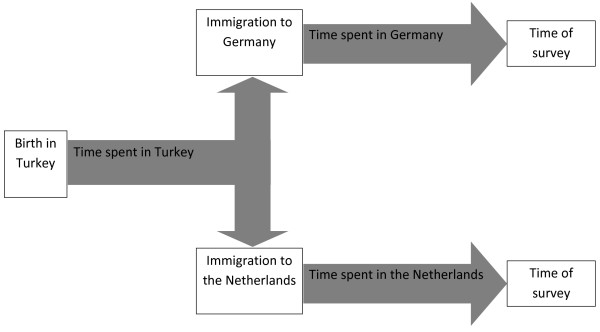
**Course of immigration among persons from Turkey now residing in Germany and the Netherlands.**

The aim of our study is (I) to compare the smoking behaviour among Turkish immigrants between Germany and the Netherlands and (II) to analyse the smoking behaviour among Turkish immigrants within Germany and the Netherlands by duration of stay in Turkey as well as in the respective host country (see Figure 1). We use German and Dutch survey data and calculate standardised smoking prevalences as well as sex-specific logistic regression models. Both between-country and within-country comparisons are required to detect a possible adaptation in smoking behaviour among Turkish immigrants.

## Background

Both Germany and the Netherlands recruited “guest workers” from Turkey following recruitment agreements in 1961 and 1964, respectively. The work-related migration from Turks with a lower socioeconomic status had its peak in the late 1960s and early 1970s. After the oil crisis in 1973 the recruitment stopped, but family reunification continued until the late 1970s. In 1980, political conditions in Turkey resulted in a second immigration wave to Western Europe, this time of refugees and asylum seekers with a higher socioeconomic status [8, 9].

In 1993, 58% of Turkish men and 14% of Turkish women were smokers [10]. By 2011, this had declined to 42% among men and 13% among women. In middle- and low-income countries a decreasing trend can be observed among men; smoking prevalences among women, however, are still very low and remained stable over time. In high-income countries there is also a decreasing trend in smoking behaviour among men but still a slightly increasing trend among women. In Germany, 35% of the men and 25% of the women reported smoking in 2011, compared to 29% (men) and 23% (women) in the Netherlands [11].

In Turkey, unlike in Germany and the Netherlands, women still experience a strong social pressure against smoking. Moreover, whereas smoking is strongly linked to a lower educational level in Germany and the Netherlands, this is only observed among men but not among women in Turkey. Turkish women with a higher educational level are more likely to smoke than those with a lower educational level [10, 12, 13]. Turkey banned smoking in public places in 1996 while the Netherlands did so already in 1990. In Germany it was not until 2007 that a smoke-free legislation was enforced for federal facilities and public transport; however, smoking in bars and restaurants, for example, is regulated by the individual states (“Länder”) of Germany [10, 14, 15].

In Germany and the Netherlands only a few studies have dealt with smoking behaviour among migrants in general and Turkish migrants in particular. In Germany, smoking prevalence among Turkish immigrants converged to that of the autochthonous population with increasing duration of stay; within the Turkish population more men than women smoked and prevalences were higher among men with lower educational level compared to those with a higher level, whereas the opposite applied to women [16, 17]. Similarly, in the Netherlands, the smoking prevalence was higher among Turkish men than among Turkish women, with similar differences by educational level as those observed in Germany [18–21]. These socioeconomic differences are, however, not universal and depend on the immigrant group under study and the smoking patterns the group experienced in their countries of origin and host countries [22].

According to Schooling & Kuh [23], not only the individual and social perceptive but also a *temporal* perspective needs to be taken into account when analysing smoking behaviour. Migrants may virtually ‘import’ health risks and resources from their countries of origin which, in turn, may be prone to change with increasing duration of stay in the host country [24]. The changing health behaviour in the host country might be the result of an acculturation process which starts immediately after arrival in the host country [25]. Acculturation describes a complex and dynamic process through which attitudes and behaviours of people change in consequence of interactions with individuals in their new social environment [26]. The concept of acculturation is widely used in health behaviour research where it is quantified using different measures that range from indirect proxy measures (such as length of stay and proficiency in the language of the host country) to multidimensional scales [27–30]. Studies on the association between acculturation and smoking among immigrants to the US show that prevalences are very low among recent immigrants from economically less developed countries now residing in developed countries but seem to increase with duration of stay in the host country [31–38].

However, the smoking behaviour does not only depend on the time spent in the host country but also on the time spent in the country of origin [39]. For immigrants this means that current smoking patterns might be influenced by the perceived social acceptance of smoking in the country of origin as well as the adaptation towards the smoking patterns in the host country with increasing duration of stay [40]. The host country’s political efforts concerning immigration and integration may also influence a possible adaptation [41].

## Methods

### Data sources

Dutch and German survey data was analysed. For the Netherlands, the POLS-basic data was used. POLS (Dutch: Permanent OnderzoekLeefSituatie) is an annual survey of the living situation among Dutch households. Computer-assisted personal or telephone interviews are used as standard questioning techniques. POLS-basic started in 1997. The annual response rate ranged between 53% (1998) and 66% (2006). The number of participants decreased steadily between 1997 (34,439) and 2009 (9,118) [42, 43]. As the annual number of migrants from Turkey with an average of approx. 250/year was too small to obtain stable results, eight years (1997-2004^a^) were combined. There was no overlapping in the data as people were not surveyed repeatedly. Year-specific descriptive analyses revealed no divergent results compared to the combined analysis.

In Germany, the 2009 micro-census (German: Mikrozensus) was used. The micro-census is a survey conducted annually on a sample of 1% of all households (approx. 390,000 households and 830,000 individuals). Computer-assisted personal interviews are used as standard questioning technique. As with the POLS survey, there were no bilingual interviewers and translated instruments. Participating in the micro-census is obligatory. The survey consists of an annual basic programme and an additional programme which is included every four years. The latter contains additional information on health (answer voluntary) and migration (answer obligatory) among others [44]. For this study, the scientific use file of the 2009 micro-census was utilised. It is a randomly drawn 70% sub-sample of the original file including data on 489,349 individuals.

### Variables used for analyses

In the Netherlands, Turkish immigrants were identified via country of birth (Turkey). In Germany, because no information on country of birth was available, citizenship was used as the main indicator for immigrant background. Persons from Turkey were identified by means of the following: birth in Germany (no) and citizenship (only Turkish : German and Turkish (dual citizenship): only German with preceding naturalisation and Turkish citizenship before naturalisation). Lengths of stay (Turkey, Germany/the Netherlands) were determined via year of birth, year of immigration and year of survey. In both countries smokers were identified based on the question “Do you smoke?”. Those answering “yes” in the Netherlands (only one category in the original dataset) and “yes, regularly” or “yes, occasionally” in Germany (two categories in the original dataset) were considered smokers in our study.

According to the study aim, a temporal interaction variable with dichotomised length of stay (LOS) in Turkey and categorised LOS in the respective host country was generated for the final analyses. LOS in Turkey was dichotomised in the following way: 0–17 years, 18 years and longer. LOS in the host countries was categorised in three categories according to the immigration waves of Turks to both countries: 1979 and earlier, 1980–1999 and 2000–2009 (recent immigrants). For the Dutch data, the first two categories were generated (1979 and earlier, 1980 and later) as the number of Turkish immigrants was too small between 2000 and 2004 (n = 26).

Information on sex, age (in years), education, occupation and income were present in both datasets. In Germany, the International Standard Classification of Education (ISCED) was available, whereas in the Netherlands education was only available in country-specific categories. As these measures serve as background information and are not directly linked to the study aim, partial inconsistency was accepted.

### Statistical analyses

This study investigates the smoking prevalence of a population that emigrated from the same country of origin to two different host countries. The question is whether there are differences in smoking behaviour among Turkish immigrants between Germany and the Netherlands. We calculated age standardised smoking prevalences and 95% confidence intervals for Turkish immigrants of both countries by sex and binary educational level. We applied the direct age standardisation method by weighting the age-specific smoking prevalences of the Turkish immigrants with the population share in the respective age groups of the old European Standard Population. For comparison, we also calculated age standardised smoking prevalences for the non-Turkish population of Germany and the Netherlands. We also investigated whether the LOS in Turkey as well as in Germany or the Netherlands has an effect on the smoking status of Turkish immigrants. We conducted logistic regression models with smoking as binary dependent variable and the temporal interaction variable as independent variable. We have, in a previous step, also estimated the single effects of LOS in Turkey and LOS in Germany/the Netherlands. Moreover, we have calculated logistic regression models with LOS as continuous variables but because the patterns observed were similar for both regression models, we decided to present the results of the models with the categorical LOS variables in order to facilitate an easier interpretation of the results. To control for confounders, socioeconomic variables (education, occupation, income) and age (for the Dutch data also survey year) were included in the models. The study population in both countries was restricted to persons aged 18–64 years due to the limited number of adolescents and older persons. Models were calculated separately for men and women. All analyses were performed using Stata Version 12.

## Results

In the German micro-census 6,517 participants aged 18–64 years were identified as Turkish immigrants. In the Netherlands, a total of 2,106 persons from Turkey aged 18–64 years participated in the POLS surveys. For characteristics of the study populations in both countries see Table 1.

**Table 1:** **Characteristics of the study population (18–64 years) in the Netherlands and Germany**

	The Netherlands (1997–2004)			Germany (2009)		
Variable	Item	n	%	Item	n	%
**Age (in years)**	18-29	675	32.05	18-29	853	13.09
	30-44	998	47.39	30-44	3179	48.78
	45-64	433	20.56	45-64	485	38.13
	*Total*	*2106*	*100.00*	*Total*	*6517*	*100.00*
	Mean: 35.86 (SD: 10.73)			Mean: 42.00 (SD: 11.00)		
**Sex**	Men	1122	53.28	Men	3287	50.44
	Women	984	46.72	Women	3230	49.56
	*Total*	*2106*	*100.00*	*Total*	*6517*	*100.00*
**Immigration year to host country** ^**1**^				2000-2009	728	11.17
	1980 and later	1081	51.33	1980-1999	2993	45.93
	1979 and earlier	986	46.82	1979 and earlier	2556	39.22
	Missing	39	1.85	Missing	240	3.68
	*Total*	*2106*	*100.00*	*Total*	*6517*	*100.00*
	Mean length of stay: 18.25 (SD: 8.34)			Mean length of stay: 24.30 (SD: 10.94)		
**Length of stay in Turkey (in years)**	0-17	1006	47.77	0-17	2822	43.30
	18 and longer	1061	50.38	18 and longer	3455	53.02
	Missing	39	1.85	Missing	240	3.68
	*Total*	*2106*	*100.00*	*Total*	*6517*	*100.00*
	Mean: 17.62 (SD: 9.27)			Mean: 18.14 (SD: 9.25)		
**Education** ^**2**^				ISCED 1 (low)	2188	33.57
				ISCED 2 (low)	2256	34.62
	Low	1197	56.84	ISCED 3/4 (high)	1703	26.13
	High	587	27.87	ISCED 5/6 (high)	342	5.25
	Not coded^3^ or missing	322	15.29	Missing	28	0.43
	*Total*	*2106*	*100.00*	*Total*	*6517*	*100.00*
**Occupation**	Existent	1043	49.53	Existent	3457	53.05
	Non-existent	1063	50.47	Non-existent	3060	46.95
	*Total*	*2106*	*100.00*	*Total*	*6517*	*100.00*
**Net income per month** ^**4**^				Low (<900€)	2115	32.45
				Middle (900€ until <2000€)	2063	31.66
	Existent	1298	61.63	High (2000€ +)	730	11.20
	Non-existent	350	16.62	Non-existent	1198	18.38
	Not coded^3^ or missing	458	21.75	Missing	411	6.31
	*Total*	*2106*	*100.00*	*Total*	*6517*	*100.00*

^1^Only two categories for the Dutch data as number of cases between 2000 and 2004 was only n = 26.

^2^Country-specific classification of education in the Netherlands (‘low’ means only compulsory education) compared to the International Standard Classification of Education (ISCED) in Germany; ISCED levels 1 + 2 are referred to as ‘low education’, levels 3 to 6 are referred to as ‘high education’.

^3^In the Dutch survey from 1998 some information were not coded in the dataset for the stated number of participants in the table; missing information could not be retrieved.

^4^Income refers to all possible sources of income, not only to salary.

### Between-country comparison

The crude smoking prevalence among Turkish immigrants in the Netherlands was 47.1% in total, 33.6% for women and 58.9% for men. In Germany, the crude prevalences were 38.3%, 25.3% and 51.2% respectively. Age standardised prevalences among Turkish immigrants were 44.3% (total), 29.4% (women) and 56.6% (men) for the Netherlands and 37.2% (total), 23.9% (women) and 50.7% (men) for Germany. Both the overall smoking prevalence and that among women and men were higher among Turkish immigrants in the Netherlands compared to those in Germany.

**Figure 2:**
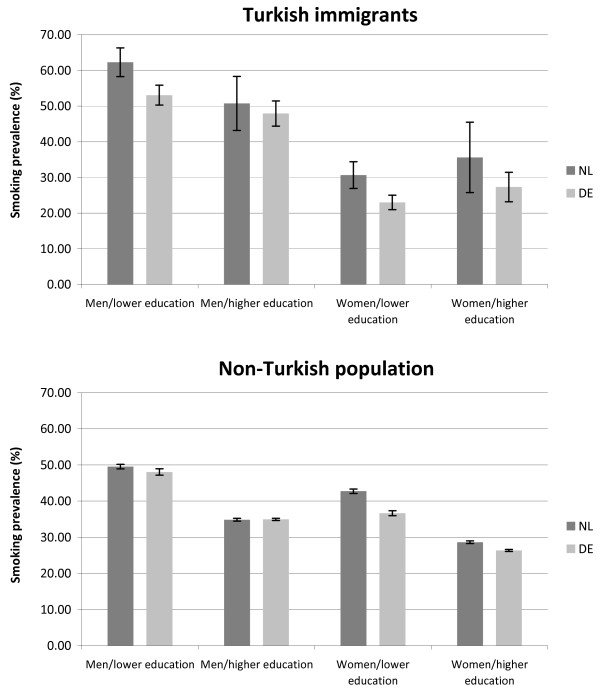
**Comparison of age standardised smoking prevalences (in%) between Turkish participants in Germany (DE) and the Netherlands (NL)/Non-Turkish participants in Germany and the Netherlands (between-country-comparison).**

The same trend became apparent when additionally stratifying the results by educational level (see Figure 2). The age standardised smoking prevalence was significantly higher among Turkish men and women with lower educational level in the Netherlands compared to their counterparts in Germany (men: 62.3% vs. 53.1%; women: 30.6% vs. 23.0%). The following pattern, in turn, was similar between Turkish immigrants in Germany and the Netherlands: there were indications of a higher smoking prevalence among less educated men compared to more educated men but a lower prevalence among less educated women compared to more educated women. Moreover, smoking prevalence was significantly higher among men than among women in both countries (not significant among higher educated persons in the Netherlands). Concerning the age standardised smoking prevalence among the non-Turkish population there were also indications of a higher smoking prevalence among persons from the Netherlands compared to their counterparts from Germany (e.g. women with low education: 42.7% vs. 36.6%; exception: men with higher education) (see Figure 2).

### Within-country comparison

The multiple logistic regression models, adjusted for education, occupation, income and age (also survey year in the Dutch data) showed differences in smoking behaviour among Turkish immigrants depending on LOS in country of origin and host country: the chance of being a smoker was lower among *Turkish men* with short LOS in Turkey (TR) and middle LOS in Germany (DE)/the Netherlands (NL) compared to those with short LOS in Turkey and long LOS in Germany/the Netherlands (NL: OR = 0.57 [95% CI = 0.36-0.89]; DE: OR = 0.73 [95% CI = 0.56-0.95]). Contrary to that, the chance of being a smoker was higher among Turkish men with long LOS in Turkey and middle LOS in Germany/the Netherlands compared to those with long LOS in Turkey and long LOS in Germany/the Netherlands (NL: OR = 1.35 [95% CI = 0.79-2.33]; DE: OR = 1.44 [95%CI = 1.03-2.02]). A similar pattern was observed for *Turkish women* in both countries: the chance of being a smoker was also lower among those with short LOS in Turkey and middle LOS in Germany/the Netherlands compared to those with short LOS in Turkey and long LOS in Germany/the Netherlands (NL: OR = 0.62 [95% CI = 0.40-0.97]; DE: OR = 0.81 [95% CI = 0.59-1.10]). Compared to Turkish women with long LOS in Turkey and long LOS in Germany/the Netherlands, the chance of being a smoker was higher among those with long LOS in Turkey and middle LOS in Germany (OR = 1.19 [95% CI = 0.82-1.74]) but lower among their counterparts in the Netherlands (OR = 0.91 [95% CI = 0.49-1.71) (see Table 2). In general, the effects for Turkish women with long LOS in Turkey were not significant and also small in both countries.

**Figure 3:**
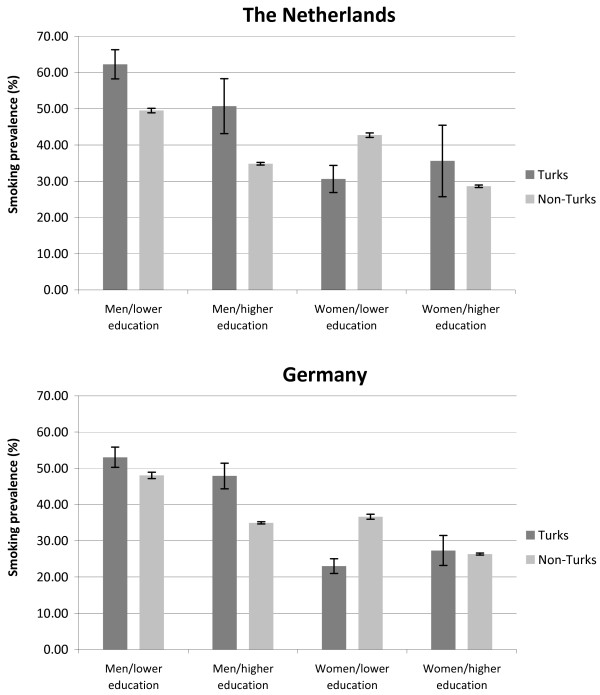
**Comparison of age standardised smoking prevalences (in%) between Turkish and Non-Turkish participants in the Netherlands/Turkish and Non-Turkish participants in Germany (within-country-comparison).**

The comparison of the age standardised smoking prevalences within Germany and the Netherlands revealed significantly higher prevalences among Turkish men than among non-Turkish men (see Figure 3). Prevalences among women with higher education indicated a similar pattern, but the difference was not significant. Among women with lower education the contrary became apparent: non-Turkish women had a significantly higher smoking prevalence than Turkish women. These patterns apply both to Germany and the Netherlands.

**Table 2:** **Binary logistic regression models for smoking among Turkish immigrants (men and women) in the Netherlands and Germany by length of stay in the country of origin and the respective host country (TR = Turkey, NL = the Netherlands, DE = Germany)** ^**1,2,3**^

**THE NETHERLANDS**						
	**Men (number of observations = 852)**			**Women (number of observations = 782)**		
**Interaction variable (length of stay in TR and NL)**	**Odds ratio**	**95% confidence interval**	**p-value**	**Odds ratio**	**95% confidence interval**	**p-value**
shortTR/middleNL	**0.57**	**0.36-0.89**	**0.013**	**0.62**	**0.40-0.97**	**0.035**
shortTR/longNL	Ref.	-	-	Ref.	-	-
longTR/middleNL	1.35	0.79-2.33	0.274	0.91	0.49-1.71	0.779
longTR/longNL	Ref.	-	-	Ref.	-	-
**GERMANY**						
	**Men (number of observations = 2383)**			**Women (number of observations = 2402)**		
**Interaction variable (length of stay in TR and DE)**	**Odds ratio**	**95% confidence interval**	**p-value**	**Odds ratio**	**95% confidence interval**	**p-value**
shortTR/shortDE	**0.22**	**0.07-0.69**	**0.009**	0.69	0.25-1.92	0.478
shortTR/middleDE	**0.73**	**0.56-0.95**	**0.021**	0.81	0.59-1.10	0.182
shortTR/longDE	Ref.	-	-	Ref.	-	-
longTR/shortDE	1.38	0.88-2.18	0.165	1.22	0.72-2.10	0.456
longTR/middleDE	**1.44**	**1.03-2.02**	**0.033**	1.19	0.82-1.74	0.358
longTR/longDE	Ref.	-	-	Ref.	-	-

^1^Short TR = 0–17 years, long TR = 18 years and longer, middle NL: immigration year 1980 and later, long NL: immigration year 1979 and earlier, short DE: immigration year 2000–2009, middle DE: immigration year 1980–1999, long DE: immigration year 1979 and earlier.

^2^All models adjusted for education, occupation, net income per month, age, survey year (only NL).

^3^Rows in bold print indicate a p-value < 0.05.

## Discussion

The most important findings of this study were that (I) the smoking prevalence among Turkish immigrants living in the Netherlands is higher than among their counterparts in Germany (irrespective of sex, age and educational level) and that (II) clear differences in smoking behaviour depending on LOS in Turkey and LOS in the respective host country were observed both within Germany and the Netherlands.

A possible reason for the higher smoking prevalence among Turkish immigrants living in the Netherlands compared to those living in Germany can be found in the prevalence among the non-Turkish population. Contrary to the estimates from the WHO [11], our analysis indicates a higher smoking prevalence among the majority population of the Netherlands compared to that of Germany. This might be partly due to differences in data sources, survey periods and questioning techniques. However, the differences in patterns between the two countries that we observed are reflected by the respective lung cancer rates, which are indicative of past smoking behaviour. Age standardised incidence (1990–2002) and mortality (1990–2010) rates for lung cancer are again higher among the Dutch than among the German population for both men and women [45]. It seems that Turkish immigrants adapted to the (different) smoking behaviours of the majority population of their respective host country. Such an adaptation might explain the observed differences in smoking behaviour among a population that actually emigrated from the same country of origin to two countries with a different smoking behaviour. However, immigrants are not only influenced by the majority population of the host country but might also themselves influence the majority population's health behaviour [46]. Acculturation is an interactive process: for example, dietary habits or leisure-time activities from the culture of the country of origin may also find their way into the culture of the host country. Consequently, adapting to the culture of the host country does not necessarily mean that one has to give up the culture of the country of origin [27–30]. Such a unidimensional concept of acculturation – merely ranging from a weak to a strong adaptation towards the culture of the host country – does not satisfy the perception of acculturation as a reciprocal phenomenon.

The tobacco policies in Germany and the Netherlands might also influence smoking patterns (but our study was not designed to test this). According to the Tobacco Control Scale by Joossens & Raw [15] for the year 2013, the Netherlands is ranked 13^th^ and Germany is ranked 33^rd^ out of 34 countries (higher positions indicate a more successful tobacco control policy). Other authors have also stated delays in acting on tobacco in Germany [47, 48]. Assuming that these political efforts had an influence on the smoking behaviour of Turkish immigrants, we would expect the smoking prevalence to be lower among Turks in Netherlands than in Germany. As the contrary is the case, tobacco policies appear to have little influence on the smoking behaviour of Turkish immigrants – it rather seems to be the collective, everyday life between the individuals that influences and shapes their smoking behaviour.

As current smoking patterns might not only be influenced by the situation in the host country but also by that in the country of origin, the differences in smoking behaviour among Turkish immigrants depending on LOS in Turkey and LOS in the respective host country are another important finding. Only one study so far has investigated the association between smoking behaviour and LOS in Germany where trends of convergence in smoking prevalence that differed between men and women were observed: whereas the smoking prevalence among Turkish men decreased with increasing duration of stay and converged to that of German men, the prevalence among Turkish women increased and also converged to that of German women [16]. Reiss et al. [49] observed these trends of convergence by sex also among the second largest migrant group in Germany, ethnic German immigrants from the former Soviet Union. In the study presented here, the LOS in the country of origin was additionally included to quantify its influence on the smoking behaviour.

In both Germany and the Netherlands the smoking prevalence seems to increase with increasing LOS in the host country and short LOS in the country of origin and to decrease with increasing LOS in the host country and long LOS in the country of origin. On the one hand this finding might – again – indicate an adaptation towards the host country’s smoking behaviour with increasing LOS among those who have spent less than 18 years in Turkey. On the other hand it also reflects an ‘imported’ smoking pattern among those with long LOS in Turkey and short/middle LOS in the host country. Unfortunately, no clear conclusion can be drawn regarding women with long LOS in Turkey. Although reasons for these findings remain speculative, there is a clear variation in smoking behaviour depending on a temporal factor: the time spent in Turkey and the time spent in the respective host country. Therefore, there are clear indications for the importance of taking a life-course perspective on smoking behaviour. This is also suggested by Schooling & Kuh [23]: health behaviour is subject to constant development and change during the life course; socio-economic and socio-cultural factors in childhood may, for example, influence the uptake and maintenance of certain health behaviours which can then be tracked until adulthood [50, 51].

### Strengths and limitations

Concerning data quality, it can be assumed that the migrant population in the micro-census is representative for the migrant population in Germany as participation in the survey is compulsory. In the Netherlands it is not obligatory to participate in the POLS. Migrants experiencing difficulties with the language of the host country are more likely to refuse study participation [52, 53]. As language difficulties are often linked to a lower socioeconomic status, the Dutch data may be biased towards an overrepresentation of people with good Dutch language skills and a higher socioeconomic status. However, *within* this study there were only slight differences in terms of occupation, education, and income between Turks in Germany and the Netherlands (see Table 1). Still, persons of Turkish origin are not a homogeneous group. In this study no information was available for example on the region of origin, immigration reasons, religion etc. – variables which might act as confounders. While comparability of the Turkish populations living in Germany and the Netherlands may be debatable, the distributions of socioeconomic and socio-demographic variables are similar. Furthermore, we controlled for socioeconomic differences between the populations that immigrated to Germany/the Netherlands in the different migration waves. The partial inconsistency in the categorisation of smoking in Germany and the Netherlands is a common problem in international comparative studies. However, we do not expect substantial differences between regular and occasional smokers with respect to our study question.

The inclusion of a temporal perspective and the analysis of an association with the smoking prevalence among Turkish immigrants is a strength of this comparative study. A major limitation is that only cross-sectional data was available. Hence, it was not possible to observe the smoking behaviour among the same group of Turkish migrants over several years – it is only possible to compare the smoking habits among Turkish migrants with different LOS in Turkey and in the respective host country at a certain point in time. As a result, one cannot speak of a temporal trend in the sense of a *decrease* or *increase* in smoking prevalence since it is still possible that a cohort-effect occurred: the different smoking prevalence among people with different lengths of stay might simply be the result of observing different cohorts. Thus, smoking prevalence should be studied longitudinally among the same group of people with increasing duration of stay.

Another issue is the different survey years that have been used in Germany and the Netherlands (1997–2004 vs. 2009). It might be argued that the higher smoking prevalence among Dutch participants compared to those in Germany might be attributed to a higher smoking prevalence in the past. In the Dutch data we included the survey year in all models and found no effect among Turkish men and a small effect among Turkish women in terms of a slight increase in smoking prevalence. A study from Germany has also analysed the temporal trend in smoking behaviour between 1990 and 2011. Among men a slightly decreasing trend was observed within the period of 21 years (difference between 1990 and 2011: -5.1 percentage points) and among women a slightly increasing trend was observed (difference: +2.6 percentage points). However, smoking patterns slowly decrease among women since the beginning of the new century [13]. Although a kind of temporal bias cannot be ruled out in our study, its influence is assumed to be negligible.

## Conclusions

This comparative study on smoking among immigrants from the same country of origin now residing in two different host countries generated important new findings: there are indications that Turkish immigrants *adapt* to the different smoking behaviours of the majority population of Germany and the Netherlands. Moreover, an increasing duration of stay in the respective host country might reinforce such an adaptation process among those who left Turkey at an early age. On the other hand, the results also reflect an ‘imported’ smoking pattern after immigration among those who left Turkey as adults, which – again – might be prone to change with increasing duration of stay in the host countries. Public health measures have to be tailored to recent immigrants with a short duration of stay in their country of origin to prevent them from starting to smoke in the host country. Additionally, the beneficial development of a decreasing smoking pattern among recent immigrants with a long duration of stay in their countries of origin has to be supported. Due to the distinct gender differences in smoking it might also be useful to make immigrant women and men aware of the social and cultural factors that might operate during the process of immigration and afterwards. As smoking is also a strong group phenomenon, Poonia [54] advised to additionally take into account the cultural context of smoking in the countries of origin and the stressful immigration process to the host countries. Implementing measures within migrant networks and involving important community representatives might prove successful in reducing smoking prevalences among immigrants.

The **new contribution** of the paper is that population groups that migrated from the same country of origin and now reside in different host countries adapt to the smoking behavior of the respective host country’s majority populations, even if the smoking patterns of the majority populations differ between each other. That was observed in this comparative study among immigrants from Turkey now residing in Germany and the Netherlands. Immigrants who left Turkey as adolescents are at a particularly high risk of commencing to smoke. Health care providers in the host countries should develop targeted prevention measures for this group.

## Endnotes

^a^After 2004 no information on country of birth was available.
